# Potent pairing: ensemble of long short-term memory networks and support vector machine for chemical-protein relation extraction

**DOI:** 10.1093/database/bay120

**Published:** 2018-11-06

**Authors:** Farrokh Mehryary, Jari Björne, Tapio Salakoski, Filip Ginter

**Affiliations:** 1 TurkuNLP group, Department of Future Technologies, University of Turku, Turku, Finland; 2 University of Turku Graduate School, Turku, Finland; 3 Turku Centre for Computer Science, Turku, Finland

## Abstract

Biomedical researchers regularly discover new interactions between chemical compounds/drugs and genes/proteins, and report them in research literature. Having knowledge about these interactions is crucially important in many research areas such as precision medicine and drug discovery. The BioCreative VI Task 5 (CHEMPROT) challenge promotes the development and evaluation of computer systems that can automatically recognize and extract statements of such interactions from biomedical literature. We participated in this challenge with a Support Vector Machine (SVM) system and a deep learning-based system (ST-ANN), and achieved an F-score of 60.99 for the task. After the shared task, we have significantly improved the performance of the ST-ANN system. Additionally, we have developed a new deep learning-based system (I-ANN) that considerably outperforms the ST-ANN system. Both ST-ANN and I-ANN systems are centered around training an ensemble of artificial neural networks and utilizing different bidirectional Long Short-Term Memory (LSTM) chains for representing the shortest dependency path and/or the full sentence. By combining the predictions of the SVM and the I-ANN systems, we achieved an F-score of 63.10 for the task, improving our previous F-score by 2.11 percentage points. Our systems are fully open-source and publicly available. We highlight that the systems we present in this study are not applicable only to the BioCreative VI Task 5, but can be effortlessly re-trained to extract any types of relations of interest, with no modifications of the source code required, if a manually annotated corpus is provided as training data in a specific file format.

## Introduction

BioCreative VI Task 5 challenge (hereinafter referred to as the `shared task*’*), focuses on extraction of relations between chemical compounds/drugs and genes/proteins, stated in biomedical texts (1). The CHEMPROT corpus that provides such annotations is used as the training and test data in this task. The aim of the task is to promote the development of systems for extracting such relations for use in precision medicine, drug discovery and basic biomedical research (http://www.biocreative.org/tasks/biocreative-vi/track-5/).

This shared task follows the well-established approach of pairwise relation extraction in the field of biomedical text mining. protein–protein interactions (PPI) were one of the extraction targets in a number of shared tasks and datasets. The BioCreative II and BioCreative III challenges (2, 3) focused on pure PPI extraction, while the BioCreative V CDR Task focused on chemical-induced disease relation extraction (4). The two drug–drug interaction shared tasks (DDI-2011 and DDI-2013) focused on the detection of adverse interactions between pairs of drugs (5, 6), and the Bacteria–Biotope relation extraction tasks aimed at extracting the location of bacteria from scientific web pages or PubMed abstracts (7–9). Finally, Pyysalo *et al*. (10) have preprocessed and unified five publicly available protein–protein interaction corpora (http://mars.cs.utu.fi/PPICorpora/), in order to facilitate seamless development and comparison of biomedical relation extraction methods. Among these tasks, DDI-2013 (6) has become popular for assessing the performance of relation extraction methods, mainly because it has a relatively large and challenging corpus.

We approach the BioCreative VI Task 5 challenge as a classification task where we classify each valid pair of entities as one of the annotated relation types or as a negative. We have developed three different systems to address the task. The first system relies on a rich set of features and a linear support vector machine (SVM) classifier (11). The two other systems are based on deep learning and require less feature engineering. Our shared task artificial neural network (ST-ANN) system utilizes an ensemble of neural networks, each having three long short-term memory (LSTM) chains (12), for representing the words, part-of-speech (POS) tags and dependency types (DTs) (i.e. edges in the sentence parse graph) along the shortest dependency path (SDP) connecting the two candidate entities. Our improved ANN (I-ANN) system is also an ensemble of neural networks, each having three LSTM chains for representing the words, POS tags and DTs along the SDP (similar to the ST-ANN) and a bidirectional LSTM (forward and backward chains) for learning a representation of the whole sentence and the two entities of interest in it. We have also experimented with several methods for combining the predictions of these systems, with the goal of increasing the overall performance.

We participated in the shared task with the SVM and ST-ANN systems (13). On the development set, our system combination approach outperformed the two individual systems, achieving an F-score of 61.09. On the test set, our SVM system achieved the highest result of our submissions with an F-score of 60.99. After the shared task, we have significantly improved the performance of the ST-ANN system. In addition, we have developed the I-ANN system, which considerably outperforms the ST-ANN system. Finally, by combining the predictions of the SVM and I-ANN systems, we achieved an F-score of 61.46 on the development set, with a corresponding F-score of 63.10 on the test set, 2.11 percentage points (pp) higher than our best test set submission during the shared task. Here we discuss all approaches and results in detail.

## Background

In all the aforementioned biomedical relation extraction tasks (including BioCreative VI Task 5), the named entities are manually annotated and given as known data to the participants, hence the aim is to build methods that are able to automatically detect statements of relations among known named entities in the given texts. In addition to the named entities, the training data for these tasks also include manually annotated relations, making these tasks ideal for the development of *supervised* relation extraction methods. These machine learning-based methods utilize the provided training data to train a classifier—e.g. an SVM, an ANN or a Naive Bayes classifier—capable of detecting statements of relations in texts.

According to Zhang *et al*. (14), supervised relation extraction methods can be broadly divided into three main groups: (i) feature-based methods, (ii) kernel-based methods and (iii) deep learning-based methods.

Feature-based methods extract a series of relevant features from the text in order to train a relation extraction classifier. In these methods, each entity pair is represented with a corresponding numerical feature vector that is further used for either training the classifier or for detection of the relation(s) (14). The list of features usually includes (but is not necessarily limited to) bags-of-words/lemmas/POS/DTs or their n-grams in the sentence or along the SDP. The Turku Event Extraction System (TEES) (15)—previously developed by members of our research group—is an example of such a system, using a rich set of features to build an SVM classifier. TEES achieved 62.99 F-score in the DDI-2011 task (5), 58.7 F-score in the DDI-2013 task (6) and the state-of-the-art performance (42.00 F-score) in the Bacteria-Biotope 2013 relation extraction task (8). Another example is the VERSE system, developed by Lever and Jones (16), which obtained the state-of-the-art result with an F-score of 55.8 in the Bacteria-Biotope 2016 relation extraction task (9). Similarly to TEES, VERSE also extracts a rich set of features in order to train a linear SVM, but utilizes a feature selection component for optimization. Finally, Raihani *et al*. (17) achieved the impressive F-score of 71.14 on the DDI-2013 corpus with a system utilizing lexical, phrase, verb, syntactic and auxiliary features.

Kernel-based methods use kernel functions that are able to directly calculate the similarity between two instances (i.e. two machine learning examples) to train a relation classification model (14, 18). In kernel methods, examples retain their original representation (e.g. as bag-of-words in the sentence, sentence dependency parse graph or sentence shallow parse graph) and the kernel method is able to assign a label to a given novel example by computing and comparing its similarity to all labeled training set examples (14, 18, 19). An advantage of kernel methods is that they can search a feature space much larger than could be represented by a feature-based approach, because the kernel functions can explore an implicit feature space when calculating the similarity between two examples (19). Kernel functions are usually used in conjunction with classifiers like SVM and voted perceptron (20). Several kernel functions have been suggested and applied for relation extraction. In a bag-of-features kernel approach, the words in the sentence are divided into three groups: before, between and after the two entities. Each group is further represented with a bag-of-features. Bunescu *et al*. (21) used this approach to build three subsequence-kernels for each bag, with the final kernel function being simply the sum of the three kernels, which is further used with an SVM classifier for relation classification. Another popular family of kernels are tree/graph kernels. Zelenko *et al*. (18) developed kernels capable of comparing the similarity of shallow parse trees and used them with SVM and voted perceptron classifiers for relation extraction. Culotta *et al*. (19) extended the previous work by introducing the `Dependency Tree Kernel’ for relation extraction and showed that their model outperforms bag-of-words kernel approach by 20 pp. Reichartz *et al*. (22) developed the `All-Pairs Dependency Tree Kernel’, and the `Dependency Path Tree Kernel’ and showed their kernels with richer structural features significantly outperform all published approaches for kernel-based relation extraction from dependency trees. Finally, Airola *et al*. (23) developed the `All-Paths Graph Kernel’ for biomedical relation extraction and showed that their method achieves the state-of-the-art performance on five protein–protein interaction corpora.

Feature-based methods extensively rely on natural language processing (NLP) tools (e.g. tokenizers, POS taggers, lemmatizers, syntactic parsers, etc.) and require heavy feature engineering to transform the input data into a `representation’ (i.e. a feature vector) that can lead to a successful relation classification. On one hand, feature engineering is skill-dependent and time-consuming (24), on the other hand, the errors in the NLP tools are amplified in the relation extraction systems, negatively impacting their performance (14). In contrast, the aim in deep learning approach is to `automatically learn’ efficient representations, suitable for the relation classification task at hand. Deep learning achieves this by introducing representations that are expressed in terms of other, simpler representations and allowing the computer to automatically learn complex concepts out of simpler concepts (25). For example, the concept of a sentence can be expressed by phrases, while phrases are composed of words and syntactical dependencies among them. This allows a modular design and training of a hierarchy of representations, with the root as the final representation used for a prediction task. A key feature is that lower-level representations (i.e. `embeddings’) can sometimes be pre-trained in advance, in an unsupervised fashion and with training data other than the training data available for the prediction task at hand. A successful example is pre-trained `word embeddings’, the vector representations for words in a language that are trained on millions of unannotated sentences, so that words with similar meanings have similar corresponding vectors in the vector space model (26). Several studies have shown that integrating pre-trained word embeddings into deep neural networks (DNNs) can improve the performance of downstream prediction tasks.

Deep learning-based relation extraction methods have recently outperformed feature/kernel-based methods on different corpora. For example, on the DDI-2013 corpus (6) all top performing methods are based on DNNs (24). The only exception is the feature-based system of Raihani *et al*. (17) with 71.1 F-score, on par with the recent deep learning-based methods. recurrent neural networks (RNN) and convolutional neural networks (CNN) are the two main neural structures that are extensively utilized in DNNs for achieving state-of-the-art performance in various NLP and text mining tasks, such as syntactic parsing, sentence classification, sentiment analysis, text summarization, machine translation, named-entity recognition and relation extraction. CNNs are inherently efficient in learning `local’ or `position-invariant’ features through discrete convolution with different size filters (kernels), because they extract the features based on *n*-grams of the sentences. In contrast, RNNs can directly model sequential data, such as the sequence of words in sentences (24). LSTM networks (12) and gated recurrent units (GRUs) (27) are variants of RNNs that utilize memory cells and/or gating mechanisms to deal with the vanishing or exploding gradients (28), a problem associated with RNNs which negatively impacts their training and prediction performance.

Yin *et al*. (29) have systematically compared the performance of CNNs with LSTMs and GRUs on various NLP tasks and have shown that the performance of CNN and LSTM/GRU networks are very close for relation extraction on the SemEval-2010 corpus (30). However, literature survey on the DDI-2013 corpus shows that at the moment, the top three methods on this corpus are based on RNNs with F-scores higher than those of CNN-based methods. Lim *et al*. (31) have achieved the state-of-the-art F-score of 73.5 with an ensemble of Tree-LSTMs; Zhou *et al*. (32) have achieved 73.0 F-score with position-aware attention-based bidirectional LSTM networks and multitask learning; and Zhang *et al*. (24) have achieved 72.9 F-score using hierarchical bidirectional LSTM networks. In contrast, the `dependency-based CNN’ developed by Liu *et al*. (33) has achieved 70.8 F-score, the `multichannel CNN’ developed by Quan *et al*. (34) has achieved 70.21 F-score and the `Syntax CNN’ (SCNN) developed by Zhao *et al*. (35) has achieved 68.6 F-score. According to Zhang *et al*. (24) the main reason is that the DDI-2013 corpus contains many long and complicated sentences, and compared to CNNs, RNN-based models can better learn the long-term dependence of the sentence that is crucial for capturing the lexical and syntactic features in the long and complicated sentences. Since CNNs work based on *n*-grams, they can encounter problems in learning from long sentences or sentences that have important clues lying far away from each other. However, we highlight that the performance of a neural relation extraction system does not boil down only to the neural network architecture it uses, but also the inputs it receives, the feature set that it uses and the training and optimization procedures that are used to train the system.

On the CHEMPROT corpus, the highest F-score (64.10) in the shared task has been achieved by Peng *et al*. (36), with a system combination approach. Their method is an ensemble of three separate systems: (i) a CNN-based relation extraction system that receives the sentence sequence and the SDP sequence as inputs, (ii) an RNN-based system that utilizes a bidirectional LSTM network to learn from the full sentence sequence and (iii) an SVM-based system that generates features based on the full sentence and SDP. This suggests that combining the power of neural models with feature-based methods is a promising approach for relation extraction. We also participated in the shared task with a system combination approach, utilizing an SVM-based system and a deep learning-based system (13). After the shared task we have improved our neural network models, hence improving our best F-score by 2.11 pps.

## Data

The CHEMPROT corpus is a pairwise relation dataset. All entities are given as known data to the participants, thus the task is to predict the relations for valid pairs of these entities. The relations are directed, always connecting a GENE-type entity (gene or protein) to a CHEMICAL-type entity. A large set of distinct types are used for annotating the relations, but these types are combined into 10 groups that are used as the actual classes for this task. Further, only five of these classes are taken into account in the task evaluation. The micro-averaged F-score of the five target classes is the official metric used for evaluation.

Cross-sentence relations constitute less than 1% of the total relations in the CHEMPROT training set. In addition, only 10 pairs in the training set have been labeled with multiple relation types. Hence, we formulate the task as a multi-class classification task where we classify each valid pair of entities as 1 of the 10 annotated relation types or as a `negative’, and we only focus on candidate pairs belonging to the same sentence.

## Methods

We develop three different systems capable of extracting relations between CHEMICAL and GENE entities. Our first system relies on a rich set of features and a linear SVM classifier (11). The two other systems are based on deep learning and require less feature engineering. Our ST-ANN system has been developed during our participation in the shared task, whereas we have developed the I-ANN system after the shared task. We also combine predictions of the SVM classifier with either ST-ANN or I-ANN predictions to boost the F-score, using a simple algorithm that is optimized on the official development set. In this section we discuss the details of each approach.

### Preprocessing

We use the TEES system (15) to run a preprocessing pipeline of tokenization, POS tagging and parsing. We convert the CHEMPROT corpus into the Interaction XML format native to the TEES preprocessing system. We test different parses generated using the TEES preprocessor wrappers for the BLLIP, Stanford converter and SyntaxNet parser software (37–39). The default parsing pipeline in our experiments consists of BLLIP constituency parsing with the biomedical domain model of McClosky (40), followed by conversion to dependencies using the Stanford conversion tool (38). We test different variants of the Stanford dependencies (SDs) representation, with the `CCprocessed’ variant being the default unless otherwise stated.

The training data incorporates 10 different types of relations, five of them being evaluated in the task. We also define and add a `negative’ type for the cases where no relation exists between the two candidate entities. Hence, we formulate this relation extraction task as an 11-class classification problem.

### SVM-based system

The SVM-based system used in this work is the TEES (15). The system is applied as is, with no task-specific modifications. The TEES system uses the SVM^multiclass^ software as the multi-class classifier implementation (41).

The TEES system has primarily been developed for the detection of `events*’* (42), a more complex alternative to pairwise relation annotations like those used in the CHEMPROT corpus. Events consist of a trigger word (usually a verb) and 0-n directed arguments that can be named entities like proteins, but also other events. In this manner, events form a complex graph where the named entities and triggers are the nodes, and the event arguments are the edges. For detecting events, the TEES system is built as a pipeline of consecutive classification steps. In the first step (entity detection), each word token in the sentence is classified as a trigger or not. In the second phase (edge detection), directed edges are predicted between all valid pairs of entities. Since multiple, different events can use the same word token as their trigger, the third step (unmerging) is used to `pull apart’ such nodes by classifying all valid trigger and argument combinations as real events or not. The fourth step (modifier detection) can be used to detect binary modality modifiers annotated for some events, such as negation and speculation.

TEES can also be used for pairwise relation extraction tasks such as the DDI (drug–drug extraction) challenges (43) or in the current work, the CHEMPROT task. In such tasks, only the second step (edge detection) of the TEES pipeline is used. The set of nodes consists of the given named entities, and relations are predicted for all valid node pairs; in the case of CHEMPROT all pairs where the first entity is of type CHEMICAL and the second of type GENE.

For all the steps in the classification pipeline, TEES relies on a rich feature representation. While most features for relation detection are generated from the shortest path of dependencies between the two entities, dependency chains outside this shortest path, bags-of-words and the linear order of tokens are also used for generating features, in an attempt to capture more of the sentence context outside the direct relation between the two entities of interest.

We test several different forms of parsing and variations of predicting the CHEMPROT corpus with TEES, but find that none of these improve performance over the default approach (see the Shared task results section for detailed information). In addition we tried using the DrugBank dataset (44) as additional features. For the three CHEMPROT corpus representations, the TEES system is trained with either the default of all 10 classes, with all the non-evaluated classes merged into a single class or with the non-evaluated classes entirely removed. For parses, we try the BLLIP parser with the McClosky biomodel and with all five types of Stanford conversion, as well as the SyntaxNet parser.

### ST-ANN system

ST-ANN is a deep learning-based relation extraction system that requires less feature engineering than the SVM system and is centered around three main ideas: (i) utilizing LSTM networks instead of simple RNNs, (ii) focusing on the words along the SDP and (iii) using an ensemble of neural networks (with identical architectures) instead of a single neural network, to stabilize the variance in the performance caused by the random initialization of the network weights.

**Figure 1:**
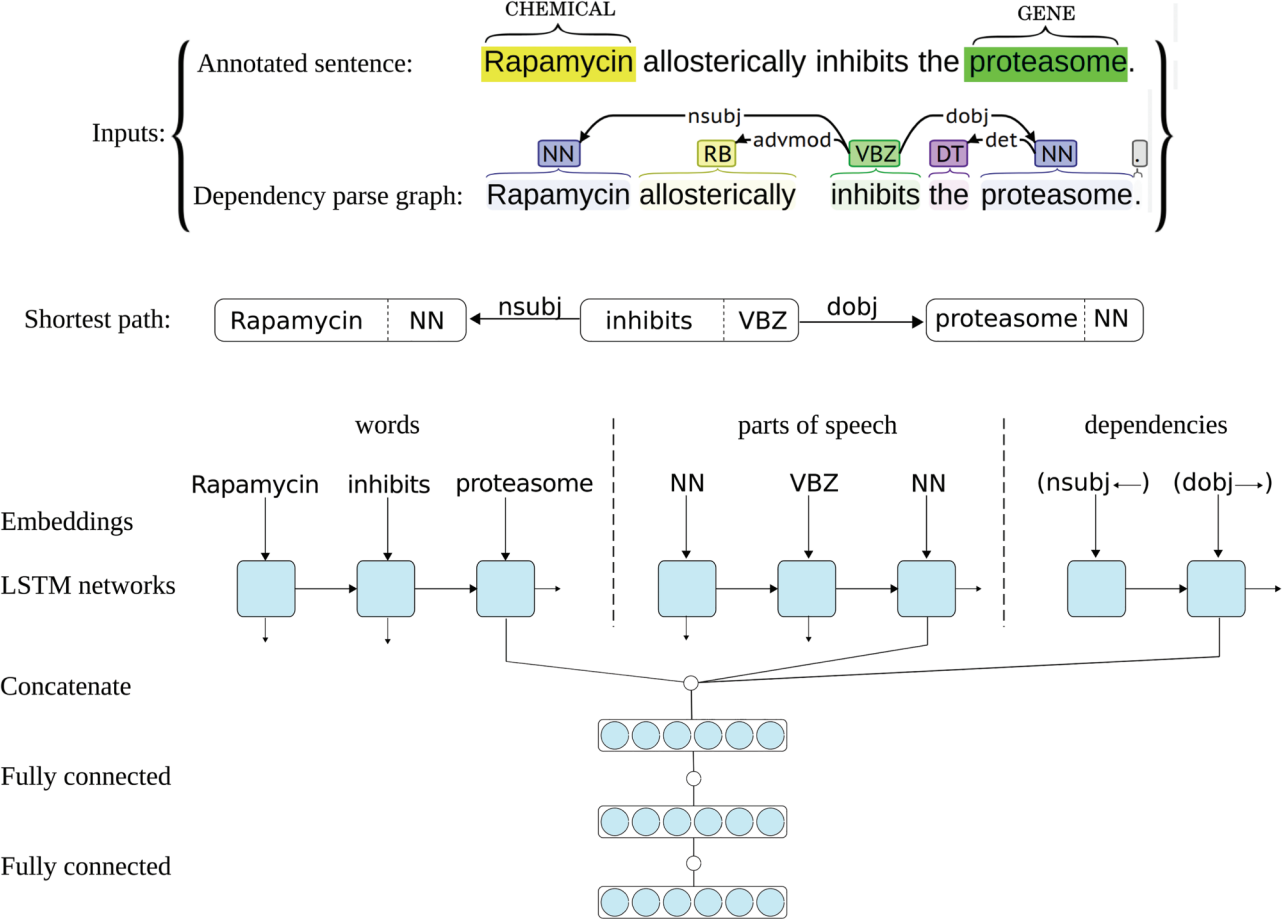
Architecture of one neural network in the ST-ANN ensemble. The figure illustrates the architecture of one neural network in the ST-ANN ensemble with an example sentence from the CHEMPROT training set and its dependency parse graph as the inputs. The shortest dependency path which connects the two entities (“Rapamycin” and “proteasome”) in the parse graph is first discovered. The path is traversed from the CHEMICAL entity to the GENE entity, producing the sequence of words, the sequence of POS tags, and the sequence of dependency types (edges) along the path. The words, POS tags and dependency types are then mapped into their corresponding vector representations using embedding lookup layers and then input to three separate LSTM chains. The outputs of the last LSTM units of the three chains are concatenated together and the resulting higher dimensional vector (i.e. the SDP vector representation) is input to a hidden dense layer. The hidden layer finally connects to the decision (classification) layer, which has a softmax activation.

The ST-ANN system has an architecture similar to the successful system we have recently developed for extracting bacteria–habitat relations from biomedical texts (45), but the predictions of the networks in this ensemble are aggregated using a different approach, more suitable for multi-class classification. Each neural network in the ST-ANN ensemble utilizes three separate LSTM chains for representing the words, representing the POS tags and representing the DTs (i.e. edges in the parse graph) along the SDP that connects the two entities. Figure 1 shows the architecture of one neural network in the ST-ANN ensemble with an example sentence from the CHEMPROT training set and its dependency parse graph as the inputs.

Even though standard RNNs are theoretically efficient sequence learning models, they usually suffer from the vanishing or exploding gradients problem (28): if the network is deep, during the back-propagation, the gradients may either decay exponentially and cause the learning to become very slow or stop altogether (`vanishing gradients’); or become excessively large, and cause the learning to diverge (`exploding gradients’). To address this problem, LSTM networks (12) and GRUs (27) have been proposed based on RNNs. LSTM-based networks exploit memory cells and gating mechanisms while GRU-based models are simpler and only utilize a gating mechanism. LSTM and GRU networks are shown to be much more efficient sequence modelers compared to simple RNNs. For example, Zhang *et al*. (24) have compared standard RNNs with LSTMs and GRUs for relation extraction and have shown that their standard RNN-based model achieves 61.4 F-score on DDI-2013 corpus, whereas the equivalent GRU-based and LSTM-based models achieve 72.4 and 72.9 F-score, respectively, ∼11 pps higher than the standard RNN-based model. In this work, we also use LSTM networks instead of standard RNNs, for capturing the information in the SDP.

The SDP that connects the two entities in the syntactic parse graph is known to contain most of the relevant words for expressing the relation between the two entities, while excluding less relevant and uninformative words (45). Figure 1 shows an example sentence from the CHEMPROT training set, its parse graph and the shortest path that connects the two entities. As can be noticed in the figure, the SDP for this particular example contains only the subject `Rapamycin*’* (the CHEMICAL), the verb `inhibits*’* and the direct object `proteasome*’* (the GENE), whereas less relevant words (the adverb `allosterically*’* and the article `the’) are put aside. Building a relation extraction system by focusing on the most important words (e.g. words in the SDP) can lead to good generalization for unseen data. Based on this observation, many successful feature-based and deep learning-based relation extraction methods have been developed (15, 16, 45–49). The ST-ANN system also generates the features along the SDP. For this aim, we first assume the parse graph is undirected and find the shortest path between the two entities (CHEMICAL and GENE), and always traverse the path from the CHEMICAL entity to the GENE entity, regardless of the order of the entity mentions in the sentence. Based on experiments on the CHEMPROT development set, we notice this approach results in significantly better generalization for unseen data, compared to traversing the path from the first occurring entity mention in the sentence to the second. Besides the existing DT edges in the parse graph, we also add an artificial edge between any two adjacent words of the sentence (word-adjacency edges). As shown by Quirk *et al*. (50), this approach mitigates the parsing errors and increases accuracy and robustness when the system is confronted with linguistic variation. For instance, if the parser produces a graph with more than one connected component, adding these artificial edges to the parse graph assures the existence of a path between the two entities. We assign a distance (weight) of one to DT edges and the distance assigned to word-adjacency edges is treated as a hyper-parameter, set (to the value of 5) using the grid-search optimization procedure described later. We then generate the features based on the words, POS-tags and DTs in this path.

As Figure 1 shows, each neural network in the ST-ANN system utilizes three separate LSTM chains. The sequences of words, POS tags and DTs are first mapped into sequences of their corresponding vector representations, i.e. embeddings, by three separate embedding lookup layers and then used as input to the three LSTM chains. For words, we use 200-dimensional pre-trained word embeddings provided by Pyysalo *et al*. (51), which have been trained on the texts of all PubMed titles and abstracts and PubMed Central Open Access (PMC-OA) full text articles using the word2vec method (26), whereas POS tag and DT embeddings are initialized randomly at the beginning of the training. During the training of our system, word embeddings are fine-tuned while the randomly initialized POS and DT embeddings are learnt from scratch. The outputs of the last LSTM units of the three chains are concatenated together, the resulting higher-dimensional vector (i.e. the SDP vector representation) is fed to a fully connected hidden layer. The hidden layer finally connects to the decision layer, having an output dimensionality of 11 (corresponding to the number of classes in the dataset, plus one for the `negative’ class), with the softmax activation.

We train the aforementioned neural network on the official CHEMPROT training data and evaluate it with the official evaluation script—provided by the organizers—on the official development data. At the beginning of training, all neural network weights (except for the pre-trained word embeddings) are randomly initialized. After the training, we notice a slight variation in the measured F-score (∼1 pp) based on different initial random weights. In other words, if we repeat training the neural network with the `exact’ hyperparameter values, and over and over again, the performance of the trained models on the development set vary in the range of 1 pp. To stabilize the variance in the performance caused by the random initialization of the network weights, we train an ensemble of four neural networks (instead of a single neural network), all identical apart from the initial (random) weights, and aggregate their predictions. Each network predicts a set of confidences for each development/test set example. The final prediction for an example is generated by summing the confidences of all networks and selecting the label with the highest overall confidence. We highlight that this particular ensemble method does not automatically improve the overall F-score, but stabilizes the performance of the ensemble (regardless of the initial random weights in each network). In other words, the ensemble acts like an average neural network, but robust and indifferent to the initial random weights used to train the individual networks. Even though the 1% variation observed in the F-scores does not seem especially excessive, we use the ensemble method for the following reasons. Firstly, the ensemble method facilitates hyperparameter optimization (i.e. finding optimal values for the hyperparameters), because it ensures the same performance level can be achieved (on the development set) if the ensemble is re-trained using the same hyperparameter values. This helps to make sure the improvements with values of <1 pp in the F-score are actually due to the chosen values for the hyperparameters and not caused by a random initialization of the weights, thus allowing us to fine-tune the hyperparameters. Secondly, as we discussed previously, our relation extraction system is not specific to the CHEMPROT corpus and can be re-trained with other training data (e.g. the DDI-2013 or the Bacteria-Biotope corpora) for other biomedical relation extraction applications. Our previous experiments with similar neural network-based relation extraction methods and different corpora indicate that when the number of weights in a neural network is high and the training set is very small, the initial random state of a model can have a significant impact on the final model and its generalization performance, thus special care is needed when dealing with such datasets. For example, the Bacteria-Biotope 2016 corpus (9) contains only 524 relations in the training set. We have previously shown that the F-score on the development set of this corpus can vary up to 9 pps based on the different initial random state of the network (45). Training an ensemble of networks—instead of a single network—helps to reduce the variance when our system is trained/optimized on different corpora for different real-world applications.

We optimize the following hyperparameters with a grid search and repeating the cycle of training the ensemble (with a set of selected hyperparameters) and evaluating it on the development data: word-adjacency edge weight, dimensionality of the POS and DT embeddings, output dimensionality of the LSTMs and the dense layer, activation functions, dropout rate, learning rate and mini-batch size.

For training we use the Nadam optimization algorithm, with a learning rate of 0.002 and mini-batch size of 32, values found to be optimal by the grid search. Similarly, we apply a dropout (52) with 0.2 rate on the output of the first dense layer. The dropout is the only explicit regularization method used. Finally, we use the early stopping technique to obtain the optimal number of training epochs: the training is stopped once the performance on the development set is no longer improving, measured using the official evaluation metric.

### I-ANN system

The I-ANN system—also an ensemble of four ANNs—has been developed after our participation in the shared task. The neural networks in the I-ANN ensemble have an architecture similar to the networks in the ST-ANN ensemble (i.e. each neural model utilizes three LSTM chains for representing the sequence of words, POS tags and DTs along the SDP), but a bidirectional LSTM (forward and backward chains) is also added to the architecture for learning a representation of the full sentence and the two entities of interest in it.

A literature survey shows that in one comparison perspective, relation extraction methods can be divided into three groups: (i) the methods that only rely on the SDP, (ii) the methods that process either full sentence tokens or the entire parse graph (e.g. graph kernels or Tree-LSTMs), not explicitly targeting and extracting features from the SDP, and (iii) mixed methods that simultaneously process full sentence tokens and the SDP and generate two distinct sets of features (two vector representations) from them. Recently, the mixed methods have become popular, performing efficiently on various relation extraction datasets. For example, on the Bacteria-Biotope 2016 relation extraction corpus (9), the state-of-the-art performance has been achieved by the VERSE system, a feature-based relation extraction system developed by Lever and Jones (16) that generates features based on the word/POS tag/DT *n*-grams in the full sentence and also in the SDP, as well as features that are generated from the two candidate entities and paths around them in the parse graph. On the DDI-2013 corpus (6), an impressive F-score of 72.9 has been achieved by Zhang *et al*. (24) with attention-based bidirectional LSTM networks that process the sequence of words in the sentence, as well as the sequence of words in the SDP. They first divide the sentence into three sub-sequences: the words before the first entity mention, the words between the two entity mentions and the words after the second entity mention. The three sub-sequences and the SDP sequence are processed by four attention-based bidirectional LSTM chains that learn the representation for each sequence. The resulting representations are then processed with an upper-bidirectional LSTM network that learns the representation of the full sentence and the SDP. On the CHEMPROT corpus, the highest F-score in the shared task (64.10) has been achieved by Peng *et al*. (36) with a system combination approach. Their method is composed of three separate systems: (i) a CNN-based relation extraction system with separate convolutional layers that simultaneously learn SDP representation and full sentence representation, (ii) an RNN-based relation extraction system that utilizes a bidirectional LSTM network and max pooling to learn full sentence representation and (iii) an SVM-based system that generates features based on the SDP and the full sentence. Based on recent successful works, we believe that learning two separate representations (for the SDP and for the full sentence) increases the classification performance of the relation extraction methods. Particularly in the case of neural models, since the SDP vector representation and the full sentence vector representation are usually concatenated together and input into subsequent layers, the neural models learn how to effectively integrate these two vector representations for the relation extraction task at hand. Inspired by the work of Zhang *et al*. (24) and the CNN-based system of Peng *et al*. (36), we propose the I-ANN system that simultaneously learns SDP vector representation and full sentence vector representation. For learning the SDP vector representation, we use the same architecture of the ST-ANN system and for learning the full sentence vector representation, we use a `bidirectional’ LSTM and max pooling, similar to the RNN-based system of Peng *et al*. (36).

**Figure 2:**
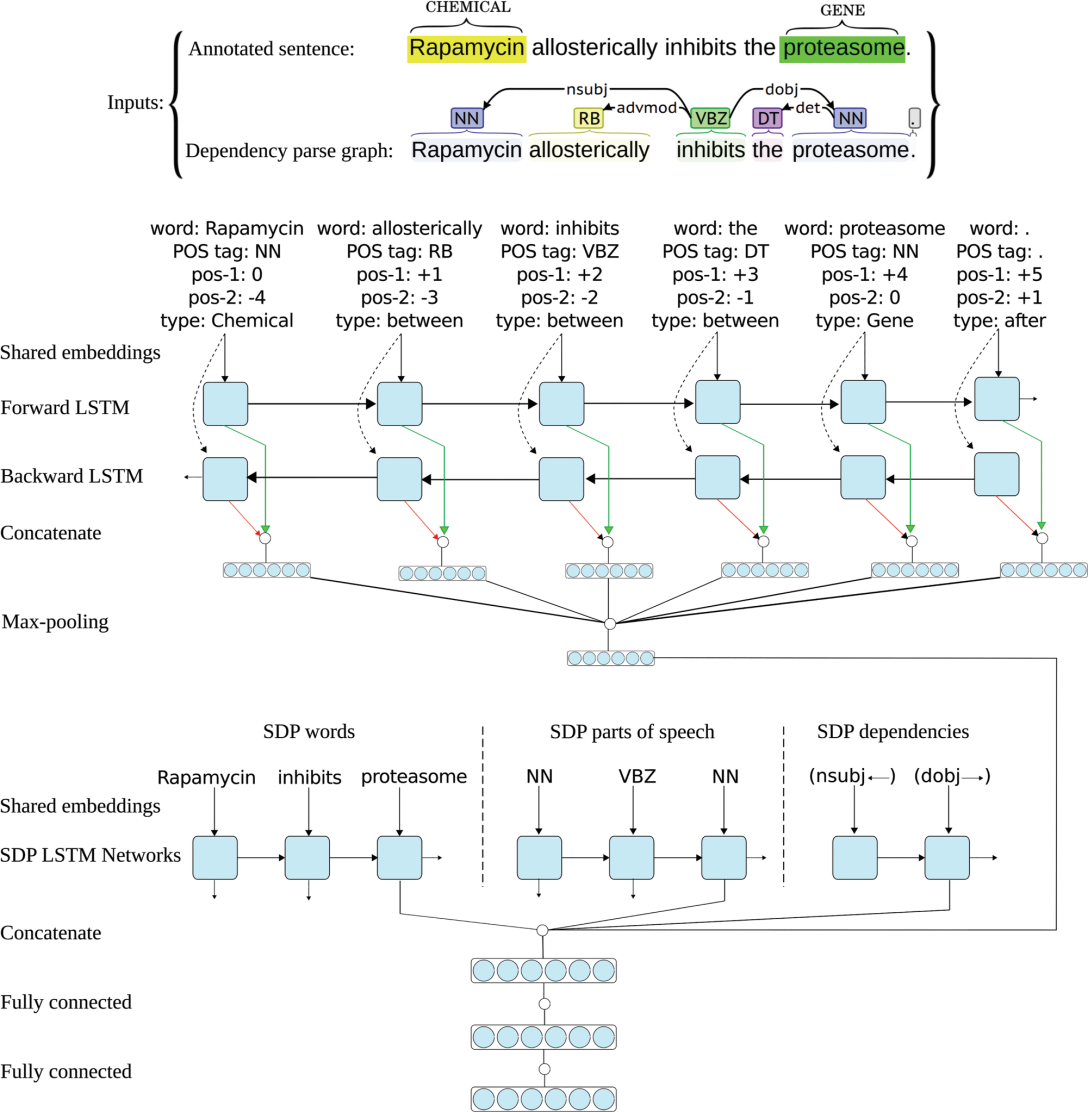
Architecture of one neural network in the I-ANN ensemble. The figure illustrates the architecture of one neural network in the I-ANN ensemble with an example sentence from the CHEMPROT training set and its dependency parse graph as the inputs. The model utilizes three LSTM chains for learning SDP vector representation and two LSTM chains (forward and backward) for learning full sentence vector representation. The words, POS tags and dependency types along the SDP connecting the CHEMICAL entity (“Rapamycin”) to the GENE entity (“proteasome”) are mapped into their corresponding vector representations (embeddings) and input to the three SDP LSTM chains. Simultaneously, for each token of the sentence, its word, POS tag, position to the first entity, position to the second entity, and token-type are mapped into their embeddings and concatenated. Forward and backward sequences of the resulting token representations are input to the forward and backward sentence LSTM chains, resulting into two hidden representation for each token (forward and backward), which are further concatenated to obtain final representations of the sentence tokens. Applying max-over-time pooling on these representations produces a vector representation for the full sentence. The outputs of the last LSTM units of the three SDP chains and the full sentence vector representation are concatenated together and the resulting higher dimensional vector is input to a hidden dense layer. The hidden layer finally connects to the decision (classification) layer, which has a softmax activation. The word and POS tag embeddings are shared among the SDP and the full sentence LSTM chains.

All RNN architectures (standard RNNs, LSTMs and GRUs) use backward connections: assuming a sentence has *n* words (*W*_1_*, W*_2_*, …, W*_t-1_*, W*_t_*, …, W*_n_), the output of the network at time-step *t* is a function of the input at time-step *t* and the output (and/or hidden state) of the network at time-step *t*-1, meaning they only capture information from the past words and the current word in the sentence. However, in many applications we want the output (i.e. representation) for a word that is dependent on whole input sequence, i.e. the context before and after that word. For example, in speech recognition, if there are two interpretations of the current word that are both acoustically plausible, we may have to look far into the future (and the past) to disambiguate them (25). For this reason, `bidirectional’ recurrent networks (53) and their variants (e.g. bidirectional LSTM/GRU networks) are introduced. In these networks, two separate RNNs simultaneously process the sequence, but from opposite directions, resulting in a forward and a backward representation for each word. The two representations for each word are further aggregated (e.g. by taking the sum or concatenation), and the aggregation is used as the final representation of the word based on the past and the future words in the sentence. As suggested by Zhang *et al*. (24) and Peng *et al*. (36), and also based on our own experiments on the CHEMPROT development data, utilizing a bidirectional LSTM (instead of a forward LSTM) results in better representations for the sentences, reflected in achieving higher performances for the relation extraction tasks at hand. Consequently, we also use a bidirectional LSTM for modeling the full sentence and the two entities in it. Figure 2 shows the architecture of one neural network in the I-ANN ensemble with an example sentence from the CHEMPROT training set and its dependency parse graph as the inputs.

As Figure 2 shows, each neural network in the I-ANN ensemble utilizes three LSTM chains for learning SDP vector representation and two LSTM chains (forward and backward) for learning full sentence vector representation. Similar to the ST-ANN system, the words, POS tags and DTs along the SDP connecting the CHEMICAL entity to the GENE entity are mapped into their corresponding vector representations (embeddings) and input to the three SDP LSTM chains. Simultaneously, for each token of the sentence, its word, POS tag, relative position to the first entity, relative position to the second entity and token type are mapped into their embeddings and concatenated. Forward and backward sequences of the resulting token representations are input to the forward and backward sentence LSTM chains, resulting in two hidden representation for each token (forward and backward), which are further concatenated to obtain final representations of the sentence tokens. Applying max-over-time pooling on these representations produces a vector representation for the full sentence. The outputs of the last LSTM units of the three SDP chains and the full sentence vector representation are concatenated together and the resulting higher-dimensional vector is input to a fully connected hidden dense layer. The hidden layer finally connects to the decision (classification) layer, which has a softmax activation.

Similar to the ST-ANN system, for words, we use the same 200-dimensional pre-trained word embeddings provided by Pyysalo *et al*. (51). It should be mentioned that these embeddings are pre-trained on the ASCII-fied PubMed and PMC-OA texts. Since some CHEMICAL/GENE entity mentions in the CHEMPROT corpus include unicode characters (e.g. IκBα, M6PRΔC, IL-1β, 11β-HSD1, AMPKα, to name a few), they do not have any corresponding vectors in the vector space model. Besides, we use only the top 1 million most frequent words from the vector space model. We thus replace all GENE entity names with the word `protein’ and all CHEMICAL entity names with the word `chemical’, if the entity name cannot be found in the model. This resulted in ∼0.5 pp increase in the F-score (when different neural network models were evaluated on the official development set).

Similar to Zhao *et al*. (35), the relative position of each token to the first and second occurring entities are first calculated and then non-linearly mapped to their corresponding 10-bit binary vectors (embeddings), where the first bit of each vector stands for the sign and the remaining bits for the distance. Additionally, inspired by the idea of `named entity embeddings*’* introduced by Peng and Lu (54), for each token of the sentence, a token type (one of the following values) is assigned accordingly:

1. If the token belongs to a CHEMICAL entity mention.
2. If the token belongs to a GENE entity entity mention.
3. If the token is located before the first occurring entity in the sentence.
4. If the token is located between the two occurring entities in the sentence.
5. If the token is located after the second occurring entity in the sentence.

The word and POS tag embeddings are shared among the SDP LSTM and the full sentence LSTM chains. During training, the pre-trained word embeddings are fine-tuned, while POS, DT, position to the first entity, position to the second entity and token-type embeddings are all learned from scratch.

Similar to the ST-ANN system, for stabilizing the variance in the performance of the I-ANN system (caused by the random initialization of the network weights), we train an ensemble of four neural networks, all identical apart from the initial (random) weights, and aggregate their predictions using the aforementioned aggregation method. Finally, we optimize the network hyperparameters by doing a grid search and repeating the cycle of training an ensemble (with a set of selected hyperparameters) and evaluating it on the development data. Table 1 shows a comprehensive list of the hyperparameters, the list of values that are tested and the optimal values that have been found and selected to build the final neural model. For example, for the dimensionality of the POS tag embeddings, we tested the values 25, 50, 75 and 100, and 25 was shown to be the best value and thus was selected to build the final model. Similarly, we tested not using a hidden dense layer at all, or using a hidden layer with the output dimensionality of 300, 500 or 1024, and it was shown that using the additional hidden dense layer with 1024 output dimensionality leads to better performance.

**Table 1:** Hyperparameters of the networks

**Hyperparameter groups**	**Hyperparameters**	**Values**	
		***Optimal value***	***Tested values***
Dimensionality of embeddings	Words	200	pre-trained
	POS tags	25	[25,50,75,100]
	DTs	25	[25,50,75,100]
	Relative position to first entity	10	Fixed size. See Zhao *et al*. (35)
	Relative position to second entity	10	Fixed size. See Zhao *et al*. (35)
	Token type	10	[10]
Output dimensionality of LSTMs	SDP words	300	[100,200,300,400]
	SDP POS tags	200	[100,200,300,400]
	SDP DTs	200	[100,200,300,400]
	Full sentence tokens	300	[200,300]
Other architecture parameters	Word-adjacency edge weight	5	[3,4,5,6]
	Hidden layer, output dimensionality	1024	[None, 300,500,1024]
	Activation functions	tanh	[tanh, sigmoid]
	Dropout rate	0.2	[0,0.2,0.3,0.4,0.5]
Learning parameters	Mini-batch size	16	[16,32,64]
	Learning rate	0.0005	[0.0005, 0.001, 0.002]

### System combination

Our SVM and the two deep learning-based systems are trained with different sets of features. This is a potential case for testing whether combining predictions of the two systems (SVM with either of ST-ANN or I-ANN) could help in achieving better performance for this task. We implement this system combination by merging the relation predictions from the two systems as either a union (OR) or an intersection (AND), resolving overlapping predictions with conflicting types using the classifier confidence scores. Since all entities are known data in this task, the predictions from the two systems can be aligned using pairs of gold standard entities.

**Figure 3:**
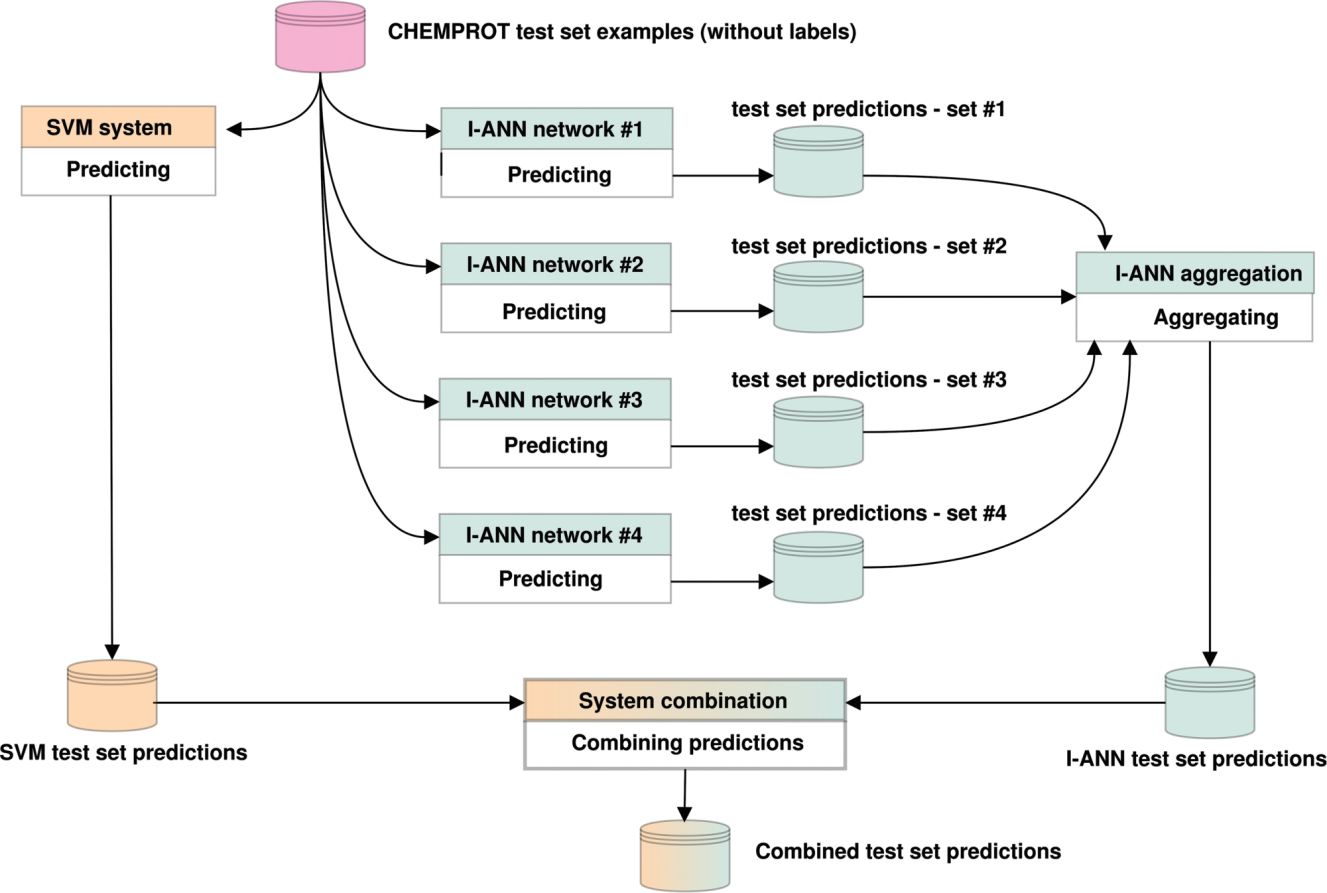
Predicting labels for CHEMPROT test set examples. The figure illustrates how the predictions of the I-ANN system and the SVM system are combined to produce a final set of predictions for the test set. Each neural network in the I-ANN ensemble predicts a set of confidences for each test set example. The confidences for each example are summed together and the label with the highest overall confidence score is selected as the relation type for that example. This aggregation procedure produces the final set of predictions by the I-ANN ensemble. The SVM system also predicts a set of confidences for each example, and the label with the highest confidence score is selected as the predicted relation type for that example. The confidence scores of the I-ANN and the SVM system predictions are further normalized into 0-1 interval. Using one of the aforementioned system combination methods (e.g. intersection or union), the two prediction sets are combined together, producing a combined set of predictions for the test set. The same procedure is applied for predicting labels for the development set/test set examples.

If only one system predicts a relation for a given pair of entities, it is either included in (OR) or discarded from (AND) the combination. If both systems predict a relation, the relation with the higher confidence score is included in the combination. Both SVM and ANN systems produce confidence scores in their own ranges. These ranges are normalized into the 0–1 interval for both systems, after which the normalized scores are compared. We experiment with combining all predictions (all 11 possible classes, including the `negative’ class), only positive predictions (all 10 possible classes) or only predictions for the evaluated classes (only the five target classes) and find that system combination in fact leads to better performance scores on the task. Figure 3 illustrates how the predictions of the I-ANN system and the SVM system are combined to produce a final set of predictions for the test set.

## Results and discussion

We conduct all of our experiments on the official development set using the official evaluation script provided by the organizers. Even though the data is annotated with 10 different relation types, the task only focuses on 5 of themby defining the official performance metric as the micro-averaged F-score of the five target classes. This is most likely due to the fact that there are much less training examples available in the data for the excluded classes. We first discuss the results of our participation in the shared task (with the SVM and ST-ANN systems and their combination) and then focus on the improved results we obtained using the I-ANN and its combination with the SVM system.

### Shared task results

In this section, we discuss all results we have achieved during our participation in the shared task. Table 2 shows the performance comparison of the SVM and ST-ANN systems, evaluated on the development data.

**Table 2:** Performance of the systems on the development set

**Evaluation on development set**	**Performance metrics**		
	***Precision***	***Recall***	***F-score***
SVM	64.55	54.72	59.23
ST-ANN	61.90	55.01	58.25
SVM + ST-ANN (OR, positive classes)	**58.45**	**63.99**	**61.09**
SVM + ST-ANN (AND, positive classes)	75.42	48.14	58.77
SVM + ST-ANN (OR, all classes)	65.82	55.55	60.25
SVM + ST-ANN (AND, all classes)	65.82	55.55	60.25
SVM + ST-ANN (OR, eval classes)	56.47	65.07	60.46
SVM + ST-ANN (AND, eval classes)	79.28	45.78	58.04

As Table 2 shows, both the SVM and ST-ANN systems have very similar performance on the task, with the SVM having an F-score 1 pp above the ST-ANN. This might be due to the fact that ST-ANN solely relies on the words and edges seen on the shortest path and we suspect that in many cases, the `trigger word’ (i.e. a token or sequence of tokens that expresses the actual relation between the two candidate entities) might be absent from this path. Consequently, the ST-ANN might not get the chance to see this information, whereas the SVM system generates features based on all tokens and dependencies near the two entities, as well as those on the shortest path connecting them. The best SVM performance is achieved with the TEES default settings, without using the DrugBank (44) features, using the BLLIP+biomodel+Stanford_CCProcessed parsing approach and including all 10 CHEMPROT relation types in the training data.

We highlight that the Stanford parsing conversion software (38) can produce five variants of the SD representation: `basic*’, `*nonCollapsed*’, `*collapsed*’, `*collapsedTree*’* and `CCprocessed*’.* As an optimization step, we tried all aforementioned conversions for the CHEMPROT corpus: we first performed constituency parsing using the BLLIP (37) with the biomedical domain model of McClosky (40), and then used the Stanford conversion tool to obtain different variants of dependency parse graphs. This resulted in obtaining five variants of the parsed corpus. For each variant, we trained the SVM system on the training data and evaluated it on the development data. The evaluation resulted in obtaining 57.13 (`basic’), 57.47 (`nonCollapsed’), 57.92 (`collapsed’), 57.82 (`collapsedTree’) and 59.23 (`CCprocessed’) F-scores. The `CCprocessed’ variant outperformed the other parsing conversion methods by ∼2 pp. Similarly, parsing the corpus with the SyntaxNet parser (39) resulted in 53.19 F-score on the development set, ∼6 pp below the best result. Consequently, we used BLLIP+biomodel+Stanford_CCProcessed variant for building the SVM, the ST-ANN and the I-ANN systems.

As Table 2 shows, for both SVM and ST-ANN systems, recall is considerably lower than precision (for instance, recall is 10 pp below precision for the SVM system). Using the OR operation in system combination considerably improves the recall (∼9 pp) while causing a comparatively lower drop in precision, leading to an ∼1–1.5 pp increase in the resulting F-score. We observe that discarding negative predictions and building the combination from all 10 positive classes result in the highest performance on the development set.

For predicting the test set, we combine the training and development data when training the SVM system. This is a common approach when using classifiers such as SVMs. However, training the neural networks on the combined data for the `optimal’ number of epochs (found during the optimization) might lead to under/over-fitting, because more/less training epochs might be needed. Finding the optimal number of epochs for training the network on the combined data is challenging. In the shared task, participating teams were allowed to submit up to 5five different test set predictions. Hence, we submitted two sets of ST-ANN predictions: (i) predictions of the ensemble of networks that are trained for 3 epochs (the optimal number found in optimization), (ii) predictions of the ensemble when the networks are trained for 4 epochs. We also combined these two sets of predictions with the SVM system predictions (using our system combination approach), resulting in a total of five sets of test set predictions. Table 3 shows the official results for our submissions on the test set, as calculated by the task organizers.

**Table 3:** Performance of the systems on the test set

**Evaluation on test set**	**Performance metrics**		
	***Precision***	***Recall***	***F-score***
SVM	66.08	56.62	**60.99**
ST-ANN (trained 3 epochs)	63.73	44.62	52.49
ST-ANN (trained 4 epochs)	63.37	43.87	51.85
SVM + ST-ANN (3 epochs)	61.05	60.06	60.55
SVM + ST-ANN (4 epochs)	60.88	59.89	60.38

**Table 4:** Performance of the systems on the development set and the test set

**Row**	**System**	**Development set**			**Test set**		
		***Precision***	***Recall***	***F-score***	***Precision***	***Recall***	***F-score***
1	SVM	64.55	54.72	59.23	66.08	56.62	60.99
2	ST-ANN	61.90	55.01	58.25	63.73	44.62	52.49
3	Corrected-ST-ANN	60.51	58.01	59.23	61.55	53.93	57.49
4	I-ANN	63.18	56.25	59.51	62.39	57.81	60.01
5	SVM + I-ANN (OR, positive classes)	58.70	63.78	61.14	61.65	66.66	64.05
6	SVM + I-ANN (AND, positive classes)	74.73	48.47	58.80	74.45	50.23	59.99
7	SVM + I-ANN (OR, all classes)	65.56	56.17	60.50	65.66	58.21	61.71
8	SVM + I-ANN (AND, all classes)	65.56	56.17	60.50	65.68	58.16	61.69
9	SVM + I-ANN (OR, eval classes)	57.65	**65.81**	**61.46**	59.05	**67.76**	**63.10**
10	SVM + I-ANN (AND, eval classes)	**79.36**	46.94	58.99	**77.79**	48.21	59.53

As Table 3 shows, compared to the development set results, our SVM system has approximately the same level of performance on the test set, achieving an F-score of 60.99, with a similar imbalance between precision (66.88) and recall (56.62). However, for the ST-ANN submissions we notice a significant drop in recall (∼11 pp) with a small increase in precision (∼1 pp), leading to an F-score of 52.49 (when the networks are trained for 3 epochs) or 51.85 (when the networks are trained for 4 epochs), which is ∼6 pp below the F-score seen on the development set. As a direct result, none of the two system combination approaches have been able to produce a result better than the SVM system alone. Hence, our best official shared task score (60.99 F-score) has been obtained using the SVM system alone.

This massive 6 pp drop in the F-score seen on the test set for the ST-ANN system is clearly abnormal. After the organizers published the test set labels, we performed a comprehensive analysis of the results of this system and discovered a critical mistake in the pipeline: the training data had not been shuffled before each training epoch, an important step preventing mini-batches of highly correlated examples. Since the neural network objective functions are non-convex, using different ordering of training samples may lead to possibly different local minima. The gradient-descent neural network training algorithms are susceptible to becoming stuck in those local minima while a better solution might exist. To summarize, shuffling training data serves the purpose of reducing variance, increasing the chance of obtaining mini-batches that are representative of the overall dataset and thus, making sure the neural network models remain general and overfit less. In the next section, we discuss how shuffling the data changed the results on the development and test sets for the ST-ANN system.

### Improved results

In this section we discuss the improved results we obtained after the shared task. Table 4 shows the precision, recall and F-score for all approaches. Row 1 (scores of the SVM system) and Row 2 (scores for the ST-ANN system, when the networks in the ensemble are trained for 3 epochs) are from Tables 2 and 3, for the sake of comparison. The Corrected-ST-ANN system (Row 3) is identical to the ST-ANN system (i.e. trained with the exact hyperparameters, including the learning rate and mini-batch size), except we have shuffled examples before each training epoch. Row 4 shows the scores achieved by our I-ANN. Note that the networks in the I-ANN systems are trained with different learning rate and mini-batch size (see Table 1), comparing to the ST-ANN system. In addition, the networks in this ensemble have been trained for 4 epochs (the optimal value based on optimization on the development set). Finally, Rows 5–10 show the scores achieved by combining the predictions of the SVM system and the I-ANN system, using various aforementioned system combination approaches. For prediction of the test set, we have combined training and development data, when training the SVM, ST-ANN, Corrected-ST-ANN and I-ANN systems.

As Table 4 shows, by comparing the scores of the ST-ANN and the Corrected-ST-ANN systems, we notice that shuffling the examples before each training epoch results in achieving ∼1 pp increase of the F-score on the development set and +5 pp on the test set. We also notice that the difference between the F-score on the development set and on the test set is much smaller for the Corrected-ST-ANN comparing to the ST-ANN system (∼2 pp vs ∼6 pp), implying that neural networks in the Corrected-ST-ANN system are more robust classification models.

The Corrected-ST-ANN system achieves the same performance level as the SVM system on the development set (59.23 F-score), but interestingly, it performs 3.5 pp below the SVM system on the test set. This suggests the Corrected-ST-ANN overfits more on the training data. Besides, the Corrected-ST-ANN system solely relies on SDP features, while the SVM system generates features based on all tokens and dependencies near the two entities, as well as those on the SDP connecting the two candidate entities. We thus investigate the scores of the I-ANN system since it utilizes whole sentence tokens, besides the features generated from the SDP.

Comparing the I-ANN system with the Corrected-ST-ANN system, we see 0.28 pp F-score increase on the development set and a comparatively larger increase on the test set (2.52 pp). This suggests that incorporating whole sentence features—besides the SDP features—into the networks in the I-ANN system actually helps achieving a better performance for the task. This is also evident as the I-ANN system achieves a similar performance level with the SVM system, both on the development set (59.51 vs 59.23 F-score) and on the test set (60.01 vs 60.99 F-score), but with the additional benefit that the I-ANN system requires much less feature engineering than the SVM system. Still, the recall is comparatively lower than the precision for the SVM, Corrected-ST-ANN and I-ANN systems.

We further investigate the potential of different approaches of combining the predictions of the SVM and I-ANN systems for achieving a higher score for the task. As Table 4 shows (Rows 5, 7 and 9), in all different possible ways of taking the union of the predictions (OR), the F-score on both development and test set improves over the SVM and I-ANN systems alone. The best F-score on the development set (61.46, Row 9) is achieved by first removing the negative and non-evaluated predictions and then taking the union of the predictions, and resolving overlapping predictions with conflicting types by using the normalized classifier confidence scores. This approach results in an F-score of 63.10 on the test set, and hence, this is our best F-score for the task, 2.11 pp higher than our best test set submission during the shared task. This is also very close to the highest F-score (64.10), achieved by Peng *et al.* (36) in the shared task.

Finally, we notice that our best approach (Row 9) also leads to the highest recall, both on the development set (65.81) and the test set (67.76), whereas removing negative and non-evaluated predictions and taking the intersection of the predictions (Row 10) leads to the highest precision of 79.36 on the developments set, and 77.79 on the test set.

### Error analysis

In this section we perform an error analysis and compare the performance of the SVM system with the I-ANN system on the CHEMPROT test set that contains 800 article abstracts. Although CHEMICAL–GENE pairs in the test set are annotated with 10 possible positive relation types, only 5 of these classes are taken into account in the task evaluation, with the micro-averaged F-score of the five target classes as the official metric for evaluation. However, since the SVM and the I-ANN systems are trained to predict and assign one of the 11 possible labels to each pair(10 positive classes and a negative class), we find it more informative to consider all predicted labels when examining and comparing the performance of the two systems.

**Table 5:** Test set annotations

**Class name**	***Evaluated in the task***	***Relation types***	***Number of annotations in the test set***
CPR:1	No	PART_OF	215
CPR:2	No	REGULATOR | DIRECT_REGULATOR | INDIRECT_REGULATOR	1743
CPR:3	**Yes**	UPREGULATOR | ACTIVATOR | INDIRECT_UPREGULATOR	667
CPR:4	**Yes**	DOWNREGULATOR | INHIBITOR | INDIRECT_DOWNREGULATOR	1667
CPR:5	**Yes**	AGONIST | AGONIST-ACTIVATOR | AGONIST-INHIBITOR	198
CPR:6	**Yes**	ANTAGONIST	293
CPR:7	No	MODULATOR | MODULATOR-ACTIVATOR | MODULATOR-INHIBITOR	25
CPR:8	No	COFACTOR	25
CPR:9	**Yes**	SUBSTRATE | PRODUCT_OF | SUBSTRATE_PRODUCT_OF	644
CPR:10	No	NOT (explicit mention of having no effects/interactions)	267
neg	No	Generated negatives for the pairs with no gold-standard annotations	10025

The test set includes 5744 positive annotations for 5665 unique CHEMICAL–GENE pairs. Even though the majority of the pairs are annotated with a single positive label, there are 79 pairs in the test set with more than one assigned label. Since both SVM and I-ANN systems predict a single label for each pair, we repeat the same predicted label for these multi-label pairs for evaluation. Besides, there are five cross-sentence annotations in the test set, but since the SVM and the I-ANN systems only extract relations from single sentences, we count these five pairs as false negatives. In addition, we generate negatives (as gold-standard relations) between any CHEMICAL and GENE entity mentions in the `same sentence’ if they do not have any corresponding gold-standard annotations. Table 5 summarizes the information about the annotations in the test set that we have used for our internal evaluation and Table 6 shows the confusion matrix, precision, recall and F-score for the SVM and the I-ANN systems. We highlight that the evaluation numbers in this section are based on the aforementioned evaluation procedure (i.e. our internal evaluation) and could not have been obtained using the official evaluation script provided by task organizers. Hence, there is a slight difference in the micro-averaged F-score of the target classes (the task metric) between the numbers reported in Tables 4 and 6, most likely due to possible differences in the evaluation of cross-sentence and multi-label pairs (duplications).

**Table 6:** Confusion matrix and evaluation metrics for the SVM and the I-ANN systems

**SVM system**		Predicted labels											**Total annotations**	**Precision**	**Recall**	**F-score**
		**CPR1**	**CPR2**	**CPR3**	**CPR4**	**CPR5**	**CPR6**	**CPR7**	**CPR8**	**CPR9**	**CP1R0**	**neg**				
True labels	**CPR1**	64	11	4	3	0	0	0	0	2	0	131	215	**70.33**	**29.77**	**41.83**
	**CPR2**	5	466	81	178	2	8	0	1	36	8	958	1743	**53.69**	**26.74**	**35.70**
	**CPR3**	0	23	295	119	3	2	0	0	3	2	220	667	**55.87**	**44.23**	**49.37**
	**CPR4**	0	58	44	1175	0	3	0	0	7	7	373	1667	**65.35**	**70.49**	**67.82**
	**CPR5**	0	14	1	19	74	3	0	0	1	3	83	198	**73.27**	**37.37**	**49.50**
	**CPR6**	0	16	0	2	4	154	0	0	1	1	115	293	**84.15**	**52.56**	**64.71**
	**CPR7**	0	7	1	5	0	2	0	0	2	0	8	25	**0.00**	**0.00**	**0.00**
	**CPR8**	0	0	1	5	0	0	0	0	0	0	19	25	**0.00**	**0.00**	**0.00**
	**CPR9**	0	10	4	28	0	0	0	0	233	1	368	644	**67.15**	**36.18**	**47.02**
	**CPR10**	0	30	17	54	0	0	0	0	2	53	111	267	**58.24**	**19.85**	**29.61**
	**neg**	22	233	80	210	18	11	0	0	60	16	9375	10025	**79.71**	**93.52**	**86.06**
**Micro-averaged F-score (all classes): 75.39**																
**Micro-averaged F-score (target classes): 60.10**																
**I-ANN system**		Predicted labels											**Total** **annotations**	**Precision**	**Recall**	**F-score**
		**CPR1**	**CPR2**	**CPR3**	**CPR4**	**CPR5**	**CPR6**	**CPR7**	**CPR8**	**CPR9**	**CPR10**	**neg**				
Truelabels	**CPR1**	108	9	1	1	0	0	0	0	5	0	91	215	**62.43**	**50.23**	**55.67**
	**CPR2**	4	731	71	139	13	11	0	0	18	14	742	1743	**39.43**	**41.94**	**40.64**
	**CPR3**	0	52	326	84	1	1	0	0	5	1	197	667	**52.84**	**48.88**	**50.78**
	**CPR4**	0	79	65	1107	2	9	0	0	35	7	363	1667	**66.65**	**66.41**	**66.53**
	**CPR5**	0	18	4	9	102	11	0	0	0	0	54	198	**60.00**	**51.52**	**55.43**
	**CPR6**	0	21	0	8	12	198	0	0	1	0	53	293	**73.33**	**67.58**	**70.34**
	**CPR7**	0	9	4	6	0	2	0	0	0	0	4	25	**0.00**	**0.00**	**0.00**
	**CPR8**	0	5	1	2	0	0	0	0	7	0	10	25	**0.00**	**0.00**	**0.00**
	**CPR9**	0	52	3	8	0	0	0	0	274	5	302	644	**56.38**	**42.55**	**48.50**
	**CPR10**	0	23	16	30	0	4	0	0	9	94	91	267	**48.45**	**35.21**	**40.78**
	**neg**	61	855	126	267	40	34	0	0	132	73	8437	10025	**81.56**	**84.16**	**82.84**
**Micro-averaged F-score (all classes): 72.15**																
**Micro-averaged F-score (target classes): 60.15**																

As Table 6 shows, both systems have failed to predict any CPR:7 (modulator) and CPR:8 (cofactor) labels, the two rarest classes in the dataset by an order of magnitude. Consequently, the F-score for these classes is zero for both systems. All pairs with CPR:7 or CPR:8 true label are misclassified as having CPR:2, CPR:3, CPR:4, CPR:6, CPR:9 relations or not having any relation (neg), by both systems.

Even though the F-score for the negative class is relatively high for both systems, all other classes are highly confused with this class. For example, about 55% (958/1743) and 43% (742/1743) of the relations having CPR:2 relation are misclassified as being negative by the SVM and I-ANN systems, dramatically dropping the recall for the CPR:2 class. This indicates that the SVM and I-ANN classifiers are not highly efficient in distinguishing positive relations from the negative ones. Building a two-step relation extraction system might be one idea to deal with this problem. These systems are generally composed of two classifiers, with the first classifier labeling each relation as being positive or negative and the second classifier detecting the type of relation for the pairs that are identified as positive. As there is high imbalance between the number of positives and negatives in the corpus, negative sub-sampling or class weighting might be other promising techniques to tackle this problem.

Considering the micro-averaged F-score of the target classes as the overall evaluation metric, the two systems have very similar performance for the task, with 60.10 F-score for the SVM system and 60.15 F-score for the I-ANN system. However, the precision and recall are much more balanced in the I-ANN system. For example, there is ∼40 pp difference between the precision and recall for the CPR:one class in the SVM predictions, whereas the difference is ∼12 pp for the I-ANN system. Similarly, precision and recall for the CPR:two classes have ∼27 pp difference with the SVM system, significantly higher that the ∼3 pp difference with the I-ANN system.

As Table 6 shows, and not surprisingly, the examples with types CPR:2 (regulation), CPR:3 (upregulation) and CPR:4 (downregulation) are more misclassified as each other, but less as other relation types (CPR:5, CPR:6, CPR:9 and CPR:10) that are semantically very different. For instance, ∼17% (119/667) of the examples with CPR:3 (upregulation) true label are misclassified as having CPR:4 (downregulation) label by the SVM system whereas only five such examples are misclassified as having CPR:5 (agonist) or CPR:6 (antagonist) relation. A manual inspection of the CHEMPROT corpus sentences revealed that the CPR:2 (regulation) is usually associated with words such as `regulation’, `interaction’, `binding’, `expression’, `relationship’, `involvement’, `change’ and `initiates’. The CPR:3 (upregulation) is associated with words/phrases such as `induced’, `promotes’, `activates’ and `increases the activity of’ and the CPR:4 (downregulation) is usually expressed with words/phrases such as `inhibits’, `blocks’ and `decreases the activity of’. However, the CPR:5, CPR:6 and CPR:9 relation types are usually expressed with semantically very different words such as `agonist’, `antagonist’, `substrate’, `catalyzes’, `mediates’ and `metabolism’.

We performed an error analysis on incorrect test set predictions made by the two systems. We noticed that in many cases that the CPR:2, CPR:3 and CPR:4 labels are misclassified as each other, the sentences are either complex, or if the sentence is simple, the SDP (or the full sentence) contains words that are usually associated with different classes. For example, in the sentence `<CHEMICAL>Xanthohumol</CHEMICAL> and 2-hydroxychalcone induced apoptosis by <GENE>Bcl-2</GENE> downregulation.’ having a CPR:4 (downregulation) interaction between the `Xanthohumol’ and `Bcl-2’ entities, the relation is misclassified as CPR:3 (upregulation) by both SVM and I-ANN systems. The SDP in this sentence (Xanthohumol, induced, downregulation, Bcl-2) contains the word `induced’ (usually associated with CPR:3) and `downregulation’ (associated with CPR:4). Similarly, there are three CPR:4 (downregulation) relations between the chemical `Cholesterol’ and the three genes in the sentence `<CHEMICAL>Cholesterol</CHEMICAL> also increases <GENE>Amyloid β</GENE>(<GENE>Aβ</GENE>) deposition and <GENE>tau</GENE> pathology.’. All the three relations are misclassified as upregulation by both systems, and the sentence contains the word `increases’, mostly associated with upregulation. In some cases, the sentences contain a mixture of positive and negative regulations, confusing the classifiers. For example, in the sentence `Although, <CHEMICAL>imatinib</CHEMICAL> primarily inhibits <GENE>tyrosine kinases</GENE>, it also stimulates the activity of <GENE>EGFR</GENE> <GENE>tyrosine kinase</GENE> in head and neck squamous tumors.’, the chemical entity `imatinib’ downregulates the (first mention of) `tyrosine kinases’, but upregulates the `EGFR’ and the second `tyrosine kinases’ entity mention. However, all three relations are assigned CPR:3 (upregulation) label by the I-ANN system.

It is also interesting to note that some examples with CPR:9 type (substrate/product of) are misclassified as having regulation, upregulation or downregulation relations. These usually belong to long and very complicated sentences that might be hard even for a non-expert human to distinguish. For example, the sentence `As discussed in this review, various progestogens including dydrogesterone and its 20alpha-dihydro-derivative, medrogestone, promegestone, nomegestrol acetate and norelgestromin can reduce intratissular levels of estradiol in breast cancer byblocking sulfatase and 17beta-hydroxysteroid-dehydrogenase type 1 activities.’ contains eight chemical entities (`progestogens’, `dydrogesterone’, `medrogestone’, `Promegestone’, `nomegestrol acetate’, `norelgestromin’, `estradiol’, `17beta-hydroxysteroid’) and two gene entities (`sulfatase’, `17beta-hydroxysteroid-dehydrogenase type 1’). All CHEMICAL–GENE pairs have CPR:4 relation (inhibitor), except (`estradiol’, `sulfatase’), (`estradiol’, `17beta-hydroxysteroid-dehydrogenase type 1’) pairs, which have CPR:9 (substrate) relation. Both classifiers have assigned CPR:4 label to all pairs and failed to detect that `estradiol’ is the substrate of the genes.

The class label CPR:10 is also very challenging since it is used for pairs with `explicit mention of not having any effects on*’* relation, which is semantically different from the negative class (`no information about having relations/interactions’). For example, in the sentence `The induction of <GENE>HO-1</GENE> by EIH was inhibited by <CHEMICAL>SB203580</CHEMICAL> but not by <CHEMICAL>SP600125</CHEMICAL>, <CHEMICAL>PD98059</CHEMICAL>, nor <CHEMICAL>LY294002</CHEMICAL>.’, the (HO-1,SB203580) pair has CPR:4 (inhibitor) relation, whereas the other three chemicals have CPR:10 relation with `HO-1’ gene. As Table 6 shows, the F-score for this class is low (29.61 with the SVM and 40.78 with the I-ANN classifier) and this class is highly confused with all other classes. For example, in the simple sentence `Neither <CHEMICAL>oxycodone</CHEMICAL> nor its metabolites activated <GENE>PXR</GENE>, <GENE>CAR</GENE>, or <GENE>AhR</GENE>.’, all three CHEMICAL-GENE pairs with CPR:10 true label are mistakenly assigned CPR:3 (upregulation) labels by our classifiers, most likely because the word `activated’ (strong indicator of upregulation) is inside the sentence. Generally, detecting such relations is a major challenge in relation extraction and while the I-ANN system performs better than the SVM system on this class, there is clearly room for improvement.

We further manually analyzed and compared the misclassifications made by the SVM system with the I-ANN system in order to check if certain syntactic/semantic patterns can be linked to only one of the classifiers. We did not find any particular patterns that can be exclusively attributed to only one of the systems. In addition, we systematically compared the two systems based on the average length of misclassified sentences (in terms of the number of tokens) to check if one system better deals with longer sentences and found out both systems have similar performance levels on long sentences.

### Comparison with other methods

In this section, we concisely compare our methods with the top performing relation extraction methods that are evaluated on the CHEMPROT corpus. Even though 13 teams participated in the shared task, only 6 teams (including us) achieved an F-score higher than 50. Table 7 lists the performance measures of the top performing methods on the CHEMPROT test set.

**Table 7:** Top performing methods on the chemprot test set

**Row**	**Method summary**	**Authors**	**Task metrics**		
			***Precision***	***Recall***	***F-score***
1	An ensemble of CNN, RNN and SVM -based systems	Peng *et al*. (36)	72.66	57.35	64.10
2	SVM + I-ANN (our best approach)	this paper	59.05	67.76	63.10
3	A deep learning-based method composed of a `pretraining’ network and a `recognition’ network, utilizing bidirectional LSTMs and CNNs	Corbett and Boyle (55)	56.10	67.84	61.41
4	An ensemble of Tree-LSTM networks	Lim and Kang (56)	67.04	51.94	58.53
5	A feature-based method with gradient-boosted trees classifier and a feature-selection component for optimization	Lung *et al*. (57)	63.52	51.21	56.71
6	Bidirectional LSTM networks	Matos (58)	57.38	47.22	51.81

As Table 7 shows, the highest F-score (64.10) in the shared task has been achieved by Peng *et al*. (36), with a system combination approach. Their method is composed of three separate systems: (i) a CNN-based relation extraction system that utilizes separate convolutional layers to simultaneously learn SDP representation and full sentence representation and uses a six-dimensional decision layer (for the five positive target classes and the negative class) with softmax activation; (ii) an RNN-based relation extraction system that utilizes a bidirectional LSTM network and max-pooling and learns full sentence representation and uses a five-dimensional decision layer (only for the five positive target classes) with a linear activation; (iii) an SVM-based system that generates features based on the SDP and tokens in the full sentence. To combine the predictions and build an ensemble of the three systems, they use either majority voting or stacking. For stacking they train a random forest classifier that uses the confidence scores (obtained from the CNN, RNN and SVM systems) as features to assign a label to each example. They build SVM, CNN and RNN models using 80% of total data (training + development) and build the ensemble using the remaining 20% of the total data. In addition, they use 5-fold cross-validation using different partitions of the data to reduce variability. Hence, they obtained five SVMs, five CNNs and five RNNs in total. For participation in the shared task, they submitted five runs (i.e. five sets of predictions for the test set), two based on majority voting and three based on the aforementioned stacking approach, each run using one SVM, CNN and RNN from one cross-validation iteration. Their best F-score (Row 1 in Table 7) has been achieved with one of the submissions based on the stacking approach.

The I-ANN system and the CNN-based system developed by Peng *et al*. (36) are similar in terms of simultaneously learning full sentence representation and SDP representation. However, the I-ANN system utilizes LSTM networks whereas their CNN model uses separate convolutional layers for this purpose. For learning full sentence vector representation, both I-ANN and the RNN-based system of Peng *et al*. (36) use a bidirectional LSTM network. The I-ANN system is trained to assign 1 of the 11 possible labels to each example, whereas their CNN/RNN models assign either one of the five target class labels or the negative label to each example. The other difference is that the I-ANN system utilizes an additional dense layer after the LSTM layers. As discussed in the previous section, these choices are made based on the optimization process we performed on the development set. We highlight that unfortunately Peng *et al*. (36) have not published the performance measures of their individual systems, thus we cannot directly compare the performance of the I-ANN or the SVM system with their individual systems. As Table 7 shows, our system combination approach and their best approach perform closely on the task, achieving 63.10 and 64.10 F-score, respectively. Their system has higher precision (72.66 vs 59.05), but lower recall (57.35 vs 67.76), compared to our system.

Corbett and Boyle (55) achieved 61.41 F-score on the task, 1.69 pp below our best approach, and 2.69 pp below the best score, with a deep learning-based approach. Their system is composed of two neural networks: a `pretraining’ network that utilizes a bidirectional LSTM network for transfer learning, and a `recognition’ network that utilizes bidirectional LSTMs and CNNs. The pretraining network—which is trained on all the titles and abstracts from PubMed records from 1809 to the end of 2015—has three inputs and two outputs. The inputs are the original sentence sequence, the `substituted’ sequence shifted one token to the right, and the `substituted’ sequence shifted one token to the left. In the substituted sequences, each token has a 50% chance of being replaced by a token randomly sampled from the lines read in that sub-epoch. The pretraining network has two outputs, one for each of the substituted shifted sequences, consisting of a sequence of numbers −1 if the token in the substituted sequence is from the original sequence, or 0 if it was randomly selected. The recognition network is trained only on the CHEMPROT data and uses the same LSTM layers that are pre-trained in the pretraining network and two additional convolutional layers and a bidirectional LSTM network. They train the pretraining and the recognition networks with series of epochs, with the first 5 epochs composed of training both networks, and the rest only training the recognition network. Although their system has a lower F-score and recall compared with our system and the system developed by Peng *et al*. (36), its main advantage is the ability to work on raw texts for extracting CHEMICAL–GENE interactions, e.g. no sentence parsing is required. Parsing is usually one of the most time-consuming steps in relation extraction system pipelines, thus eliminating this step may considerably improve the run-time performance of large-scale real-world applications.

Lim and Kang (56) participated in the shared task with an ensemble of Tree-LSTM networks (59) that process the sentence parse graph. Each node in the Tree-LSTM architecture is a word in the graph, represented by concatenating embeddings of its words, relative positions to the two entities and a subtree containment feature. The subtree containment feature for a node is simply `True*’* when one of the target entities exists in the leaves of the current node, and is `False*’* otherwise. These two values are further mapped into two 10-dimensional embeddings: if the value is `True’, every element of a vector is +1; otherwise, every element in a vector is 0. In contrast with normal LSTM networks in which each unit receives the input only from the previous unit (i.e. the previous word in the sentence or SDP), in the Tree-LSTM network, each node in the tree receives the input from multiple child nodes (leaves) and updates the hidden state of current node using those inputs. Similar to the I-ANN system, they also train an ensemble of the neural networks and aggregate their predictions by simply taking the sum of the confidence scores to deal with the variance caused by random initialization of network weights. Their method achieved an F-score of 58.53 on the task, 4.57 pp below our F-score and 5.57 pp below the highest F-score achieved by Peng *et al*. (36) during the shared task.

Lung *et al*. (57) achieved an F-score of 56.71 using a feature-based method that relies on manually engineered features, extracted from both semantic pattern and dependency parse graph of the sentence. The semantic pattern reveals whether/how chemical–protein interactions are stated in the sentence, whereas the dependency graph provides the information on how words are interconnected in the sentence. To analyze the semantic pattern, they have manually built an extensive list of words (including the `trigger’ words) and check whether these words are found in the sentence or not. In addition, they employ a set of features previously found to be beneficial for protein–protein relation extraction from biomedical texts. For example, a binary feature captures whether negative words (e.g. `not’, `incapable’ and `unable’) are in the region covered by the candidate pair and a binary feature shows if sentence breaking words (e.g `although’, `therefore’, `whereas’) exist in the region. They have reported these features are helpful in chemical–protein relation extraction as well. To analyze the sentence structure, they only target and extract a set of features from the SDP. These features include the number of tokens in the SDP, as well as binary features for checking the presence of different DT edges in the SDP. They use gradient boosted trees for classification and feature selection to optimize their system.

Finally, Matos (58) achieved 51.81 F-score on the task (11.29 pp below our F-score), using relation extraction systems composed of three to six bidirectional LSTM networks. All of their networks utilize three bidirectional LSTMs for processing the words, POS tags and DTs along the SDP. However, they also experiment with using up to three additional bidirectional LSTMs, for processing the words before/between/after the two entities. Unfortunately, a few important details are missing from their paper that are necessary for a correct comparison of their system with the ST-ANN/I-ANN systems. For example, it is not clear how the outputs of the bidirectional LSTMs are combined together in their networks (e.g. in the I-ANN system, max pooling is first applied and SDP and full sentence vector representations are further concatenated). Similarly, no details about the output dimensionality and the activation function of the decision layer in their networks is mentioned, hence it is not clear whether they assign one of the five target class/the negative label to each example, or similarly to the ST-ANN/I-ANN systems, they assign one of 11 possible labels. On the development set, they achieved 54.70 F-score (with the system that uses all six bidirectional LSTMs), 56.64 F-score (with the system that uses three bidirectional LSTMs for the SDP) and their highest F-score of 59.19 has been achieved with a system utilizing three bidirectional LSTMs for the SDP and a bidirectional LSTM for the words between the two entities. However, on the test set, the network with all six LSTMs has achieved a lower F-score of 51.81 (with ∼7 pp increase in the precision, but 14 pp drop in the recall, compared to the results on the development set). The F-score for the system with the three bidirectional LSTMs (for the SDP) has dramatically dropped to 34.18, and for the system with the four bidirectional LSTMs to 36.77. As mentioned in their paper, further error analysis is needed to give some indication on how generalization could be improved.

## Runtime performance and technical details

We implement the systems using the Python programming language (v2.7) with the Keras (60) deep learning library and the Theano tensor manipulation library (61) (as the backend engine for Keras) for implementing the neural network models. All neural network parameters not explicitly discussed in this paper were left to their defaults in Keras. All computations were run on a single server computer equipped with 64 gigabytes of memory, one 8-core CPU and one NVIDIA^Ⓡ^ TESLA^Ⓡ^*K80* GPU (with 4992 CUDA cores). Parsing and all python processing, including e.g. file manipulation, the TEES pipeline and system combination were run on the CPU, whereas all neural network related calculations (training, optimization and prediction) were run on the GPU, using the CUDA toolkit version 7.5.

The CHEMPROT corpus contains 2432 PubMed abstracts (1020 abstracts in the training set, 612 abstracts in the development set and 800 abstracts in the test set) (1). Parsing the corpus and conversion into the TEES XML format takes about 1 hour and 35 minutes. Training the SVM system takes about 40 minutes, whereas training each of the four neural models in the I-ANN ensemble takes ∼6 hours, on the combined training and development sets. These times include feature generation, but the training and development sets are already converted into the TEES XML format and parsed. Prediction of the test set using the SVM system takes ∼4 minutes, and with each of the four neural networks in the I-ANN ensemble, ∼6 minutes. Aggregating the predictions of the networks and running the system combination code takes less than a minute. Consequently, the prediction time for each abstract is in average ∼2.2 seconds, assuming that the abstract has already been parsed.

We highlight that the number of neural networks in the I-ANN system is implemented as an input parameter in our software, with 4 being the default value and 1 as the minimum possible value. Hence, training an ensemble is optional. However, because of the reasons discussed earlier, we recommend to train an ensemble of networks when training/optimizing our system on a new corpus, if sufficient computational resources are available.

## Conclusions and future work

In this study, we presented three different systems capable of extracting relations between CHEMICAL and GENE entities, as a part of our participation in the BioCreative VI Task 5 (CHEMPROT) challenge. Our SVM system relies on a rich set of features generated from all tokens and dependencies in the SDP and near the two candidate entities. Unlike the SVM system, our ST-ANN and I-ANN systems are based on deep learning and require less feature engineering. The ST-ANN system solely relies on the SDP features, whereas the I-ANN system utilizes features generated from the whole sentence, besides the features generated from the SDP. The ST-ANN system has lower performance compared to the SVM and I-ANN systems, while the I-ANN and SVM perform equally well on the development and test sets, suggesting that incorporating features gathered from the full sentence actually helps achieving better scores for the task, regardless of the classification method.

We also experimented with basic methods of combining the predictions of the SVM and either the ST-ANN or I-ANN systems (e.g. taking the union/intersection of the predictions of two systems) and noticed system combination achieves the highest F-score of 63.10 on the test set, 2.11 pp higher than our best test set submission during the shared task. Our best F-score is 1 pp below the highest score achieved by Peng *et al*. (36) in the shared task.

There are many interesting future directions that we would like to explore. As we discussed in the previous section, the SVM and I-ANN systems are not highly efficient in distinguishing positive examples from the negative examples, i.e. the examples of other classes are highly misclassified as being negative, lowering the recall of non-negative classes. We would like to investigate whether two-stage classification and/or negative sub-sampling or class weighting can diminish this problem.

Although the ensemble method we used in the I-ANN system addressed the problem of variance in the performance (caused by random initialization of network weights), it does not improve the overall F-score, because the ensemble acts like an average neural network, but robust and indifferent to the initial random weights used to train the individual networks. We would like to try better ensemble methods. One idea is to train the ensemble but instead of taking the sum of the `all’ networks confidences, we take the sum of *N* top-performing networks. Even though this approach might seem to be very promising, it can lead to heavy overfitting on the development set and consequently, poor generalization for unseen data. We think that heavy regularization and/or cross-validation [as used by Peng *et al*. (36)] will be necessary in that case. In addition, in this work we used basic methods (e.g. taking union/intersection) for combining the predictions of the SVM and I-ANN systems. As a future work, we would like to investigate better system combination approaches [such as the stacking approach used by Peng *et al*. (36)], and see to what extent the overall performance of our method can be improved.

Even though we used pre-trained word embeddings for the I-ANN system, other embeddings (e.g. the POS tag and DT embeddings) were initialized randomly and learnt from scratch. Similar to using pre-trained word embeddings, we would like to investigate whether pretraining the other embeddings can improve the performance of the I-ANN system. One idea is to train the I-ANN system on other biomedical relation extraction corpora (such as DDI-2013) and use the learnt embeddings to train/optimize the I-ANN system on the CHEMPROT training data.

Additionally, we would like to investigate different methods of incorporating the information in the whole parse graph into the neural networks. Although the I-ANN system utilizes the words/POS tags/DTs in the SDP, other word-dependencies far outside the SDP can play a critical role, considerably affecting the meaning of the relations expressed in the sentence. As discussed in the error analysis section, in the simple sentence `Neither <CHEMICAL>oxycodone</CHEMICAL> nor its metabolites activated <GENE>PXR</GENE>, <GENE>CAR</GENE>, or <GENE>AhR</GENE>.’, all CHEMICAL–GENE pairs with CPR:10 true label were mistakenly assigned CPR:3 (upregulation) labels by our classifiers. In this sentence, the key negation words (`Neither’ and `nor’) are not part of the SDP connecting the CHEMICAL to the GENE entities, and most likely because the word `activated’ (strong indicator of upregulation) is in the sentence, the relations are misclassified. One approach for incorporating the whole parse graph into neural networks is the Tree-LSTM neural architecture (59), used by Lim and Kang (56) for relation extraction. Unfortunately, they achieved 58.53 F-score on the task, 4.57 pp below our F-score. We would like to investigate whether/how the Tree-LSTM network architecture can be modified to obtain higher scores for the task. Finally, we want to explore different possible ways of incorporating DT *n*-grams (i.e. `paths*’* in the sentence parse graph) into neural networks as embeddings for this aim.

We highlight that the systems we presented in this study are not applicable only to the BioCreative VI Task 5 and can be effortlessly re-trained to extract any types of relations of interest, with no modifications of the source code required, if a manually annotated corpus is provided as training data in the Interaction XML format (15).

## Notes

**Database URL:** https://github.com/TurkuNLP/BioCreativeVI_CHEMPROT_RE
